# Oligosaccharide model of the vascular endothelial glycocalyx in physiological flow

**DOI:** 10.1007/s10404-018-2037-5

**Published:** 2018-01-29

**Authors:** Maria Pikoula, Matthew B. Tessier, Robert J. Woods, Yiannis Ventikos

**Affiliations:** 1 Department of Engineering Science, Institute of Biomedical Engineering, University of Oxford, Oxford, OX1 3PJ UK; 2 Farr Institute, UCL Institute of Health Informatics, 222 Euston Road, London, NW1 2DA UK; 3 Complex Carbohydrate Research Center, University of Georgia, 315 Riverbend Road, Athens, GA 30602 USA; 4 Department of Chemistry, University of Georgia, 140 Cedar St, Athens, GA 30602 USA; 5 Department of Mechanical Engineering, University College London, Torrington Place, London, WC1E 7JE UK

**Keywords:** Glycocalyx, Molecular dynamics, Mechanotransduction, Flow

## Abstract

Experiments have consistently revealed the pivotal role of the endothelial glycocalyx layer in vasoregulation and the layer’s contribution to mechanotransduction pathways. However, the exact mechanism by which the glycocalyx mediates fluid shear stress remains elusive. This study employs atomic-scale molecular simulations with the aim of investigating the conformational and orientation properties of highly flexible oligosaccharide components of the glycocalyx and their suitability as transduction molecules under hydrodynamic loading. Fluid flow was shown to have nearly no effect on the conformation populations explored by the oligosaccharide, in comparison with static (diffusion) conditions. However, the glycan exhibited a significant orientation change, when compared to simple diffusion, aligning itself with the flow direction. It is the tethered end of the glycan, an asparagine amino acid, which experienced conformational changes as a result of this flow-induced bias. Our results suggest that shear flow through the layer can have an impact on the conformational properties of saccharide-decorated transmembrane proteins, thus acting as a mechanosensor.

**Electronic supplementary material:**

The online version of this article (10.1007/s10404-018-2037-5) contains supplementary material, which is available to authorised users.

## Introduction

Atherosclerosis, the principal cause of heart attack and stroke, is the world’s number one cause of death and disability (Yusuf et al. 2015). Although most vascular diseases are well established as lipid-driven illnesses, recent studies suggest that inflammation and blood flow may be discrete or concomitant causes to the formation of atherosclerotic lesions (plaques) (Halliday et al. 2011) and certainly play a role in other vascular abnormalities, like aneurysms. It is hypothesised that the endothelium, a monolayer of cells that covers the lumen, is central to the development of these plaques (Gouverneur et al. 2006). This claim is supported by experiments demonstrating that endothelial cells sense flow directionality (Wang et al. 2012) to regulate processes such as gene transcription and nitric oxide release (Ensley et al. 2012), altering both the geometrical shape (Dewey 1984) and the biochemical response of the cell (Chien 2008). Indeed, the role of fluid shear stress (FSS), more widely known as wall shear stress (WSS), in vasoregulation (Pahakis et al. 2007) as well as cell differentiation (Adamo and García-Cardeña 2012) is widely recognised.

The surface of endothelial cells (ECs) is coated with a multitude of membrane-bound macromolecules which constitute the endothelial glycocalyx layer (EGL). In healthy vasculature, the EGL is the first barrier at the blood–endothelium interface and as such has many roles. It acts as a soft gel, which controls the permeability of blood-borne molecules from plasma to the endothelial surface (Henry and Duling 1999), regulates coagulation by inhibiting platelet adherence (Chappell et al. 2011) and protects against leukocyte adhesion-related inflammation (Mulivor and Lipowsky 2002) Chappell et al. (2010). Recent experiments elucidate the dynamics of the glycocalyx in the presence of flow (Koo et al. 2013) and in response to transient removal of the EGL by enzymatic treatment (Giantsos-Adams et al. 2013). Furthermore, it has been shown that the EGL plays an important role in red blood cell flow reduction in microcapillaries (Lanotte et al. 2014) as well as fluid shear stress (FSS) mechanotransduction (Florian et al. 2003; Tarbell and Ebong 2008).

The FSS transduction mechanism is believed to involve the long, negatively charged carbohydrate polymers that compose the majority of the EGL (Weinbaum et al. 2007). Two possible signal transduction mechanisms have been proposed by which these polymers convey FSS information to the ECs. According to the first hypothesis, put forward by Siegel et al. (1996), the unravelling of these long polysaccharides increases the number of Na$^+$ ion binding sites, leading to signal transduction. However, this proposed mechanism was not further explored. In the second and most prevalent hypothesis, the shear stress acting on these polymers is directly transmitted through the transmembrane proteoglycans to the actin cytoskeleton or intracellular signalling molecules (Fu and Tarbell 2013). The cell membrane itself does not sense flow (as shear at the bottom of the EGL is nearly zero) but a torque, coming from the flow-induced drag force acting on the EGL fibres.

Several families of glycoconjugates (complexes between glycans and other molecules such as proteins or lipids) exist within the EGL. The principle glycoconjugates in the EGL are proteoglycans (heavily glycosylated proteins), some soluble components (such as hyaluronic acid), and glycoproteins. The glycoproteins are proteins with glycans covalently linked to the hydroxyl group of the amino acids serine, threonine, tyrosine, hydroxyproline, or hydroxylysine (*O*-glycans) or through an *N*-glycosidic linkage to asparagine (*N*-glycans). These glycans exhibit a variety of structures (high-mannose, complex, and hybrid) and can be used to model neutral systems wherein the role of flow can be studied with relatively low computational cost.

Molecular dynamics (MD) simulations have been extensively implemented in the study of biochemical pathways of cells mainly in the areas of genomics, proteomics (Karplus and McCammon 2002) and glycomics studies (Turnbull and Field 2007). The glycocalyx consistency occurring on cell surfaces is cell-type specific, as well as characteristic of the developmental stage of the cell. However, a series of glycan structure types is common to most eukaryotic cell surface environments. Among the group of glycoproteins, the so-called *N*-glycoproteins carry the *N*-glycans bound to the sidechain of an asparagine amino acid of the protein via an *N*-glycosidic linkage to *N*-acetylglucosamine, which forms the nonreducing end in all *N*-glycans.

Eriksson et al. (2008) developed an EGL model based on branched *N*-glycan octasaccharides of the high-mannose type with the purpose of investigating alterations in ion hydration and solvation properties with regard to oligosaccharide density in the MD simulations. This was a first attempt at an all-atom EGL model under static conditions (“static” in this sense referring to equivalent static culture conditions but specifically meaning conditions of diffusion). Most recently, Cruz-Chu et al. (2014) conducted large-scale MD simulations on a system inclusive of lipid bilayer, transmembrane protein and heparan sulphate in static and flow conditions. We envisage that such efforts in atomic-scale investigations will lay the foundations for describing the meso- and macroscale properties and dynamic behaviour of these complex carbohydrates.

Using Eriksson et al. high-mannose octasaccharide as a prototype for the EGL, we aim to answer two questions related to the effect of flow: (1) What is the change in the glycan conformation and orientation; and (2) what effect does flow have on the amino acid which tethers the glycan to the glycoprotein anchor? We expand on the Eriksson et al. model of a well-characterised glycan to develop a method with the aim of subsequently applying the lessons to the larger EGL proteoglycans and their associated negatively charged long-chain polymers.

As it will become more clear in the upcoming results section, the velocity of the imposed flow is high, and therefore the simulations are meant to represent large vessels, such as the aorta, femoral, carotid.

## Methodology

### Simulation parameters

**Fig. 1:**
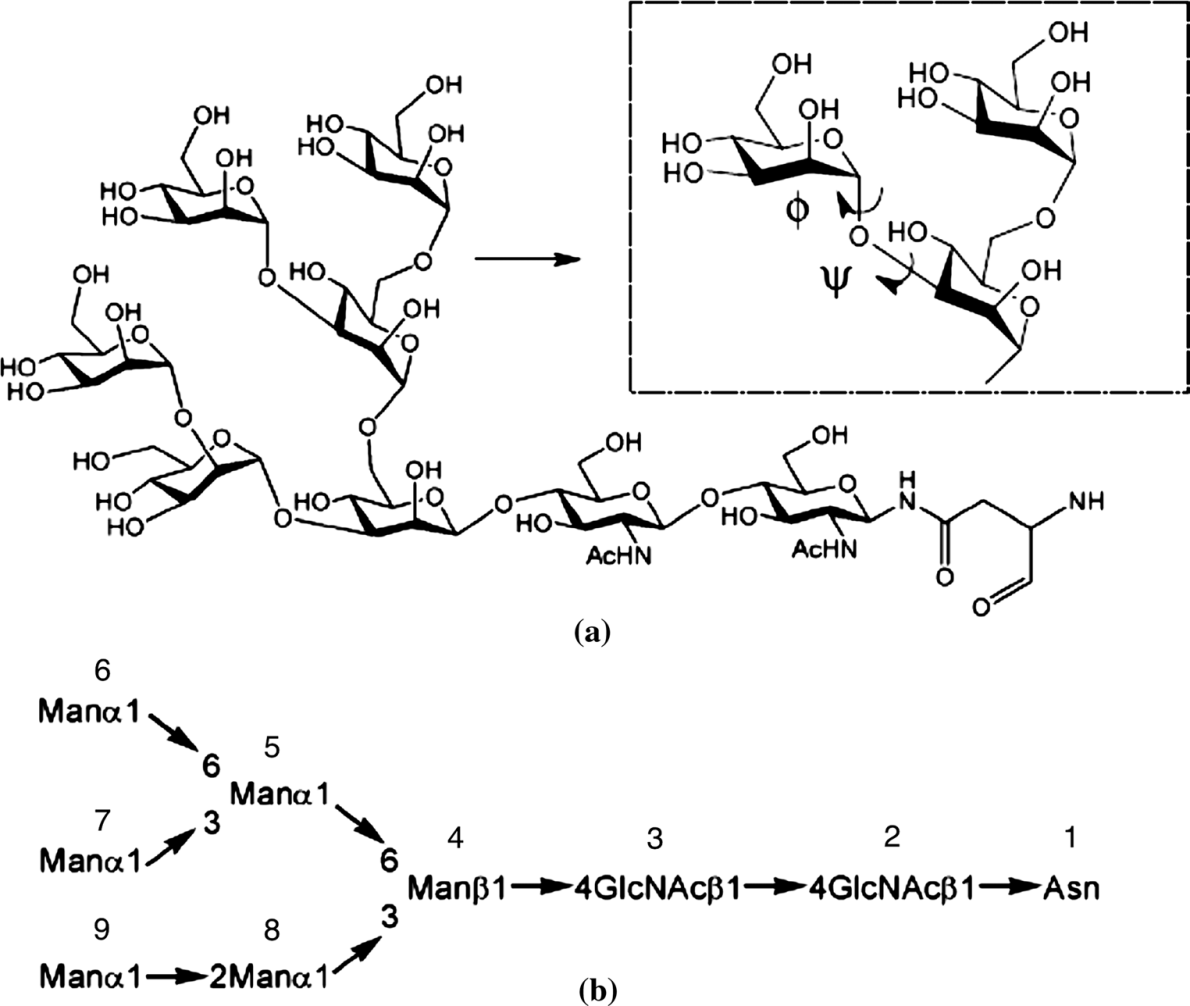
Structure of the branched asparagine-linked *N*-glycan ${Man_6GlcNAc_2}$: **a** in detail, together with a zoomed-in part showing the two dihedral angles $\phi$ and $\psi$ describing the $Man(7)(\alpha 1-3)Man(6)$ glycosidic linkage and **b** in its abbreviated form with used numbering of monosaccharide residues indicated (Eriksson et al. 2008)

The simulation set-up described below followed generally that of Eriksson et al. (2008). Due to the significant computational demands the flow implementation adds to the system, we only considered a single glycan configuration, as opposed to an array of glycans. This limitation is partially alleviated by the lateral periodic boundary conditions imposed. The asparagine-linked *N*-glycan $Man_6GlcNAc_2$ is shown in Fig. 1. Classical mechanical molecular dynamics (MD) simulations were performed on systems consisting of an isolated $Man_6GlcNAc_2$ unit in 0.15 M NaCl aqueous solution.

**Fig. 2:**
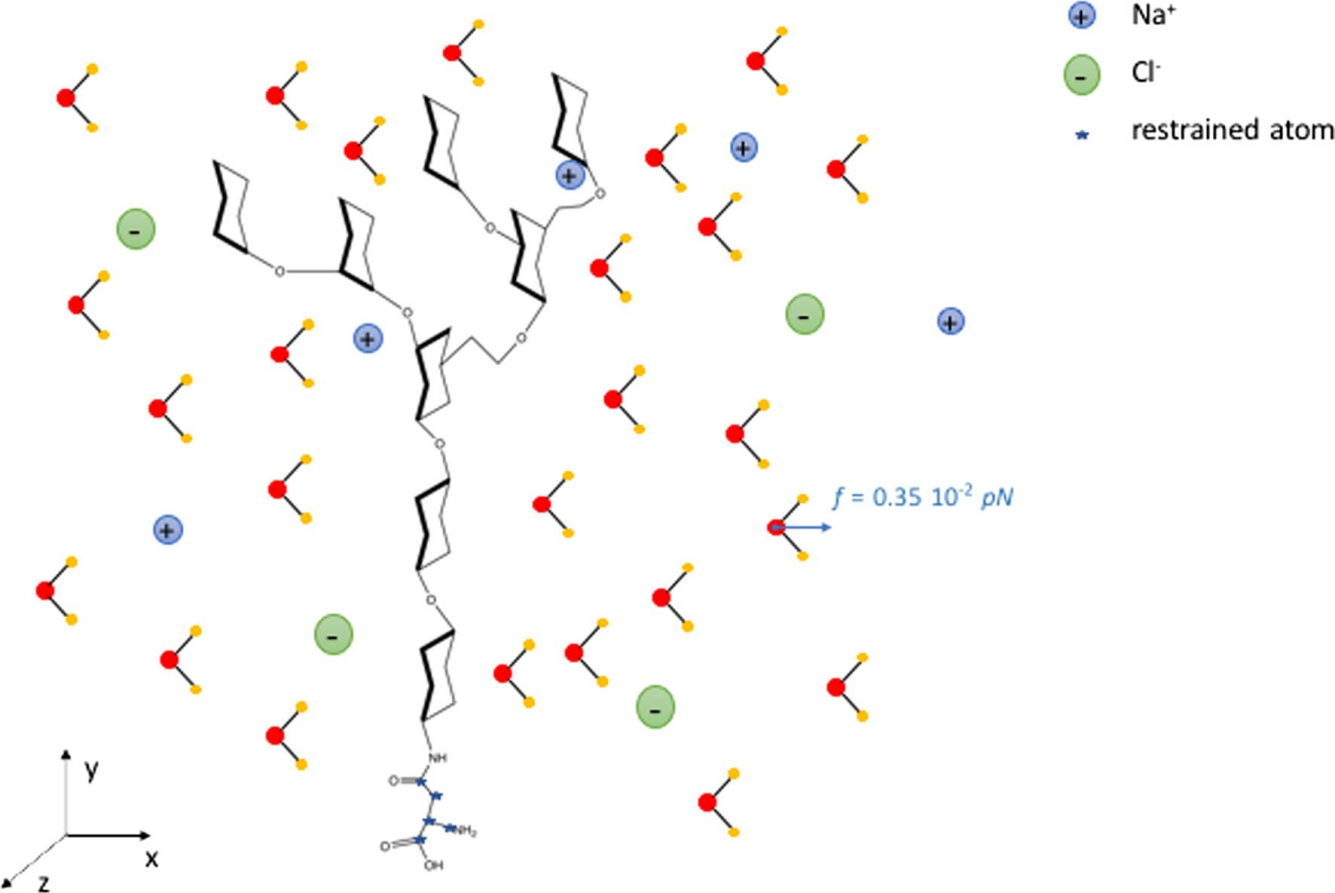
Schematic representation of the solvated ${Man_6GlcNAc_2}$ system: sodium and potassium ions are depicted in blue and green, respectively. Restrained atoms on the asparagine end are indicated with blue stars. The accelerating force and direction imposed on the oxygen atoms are also indicated (colour figure online)

The *N*-glycan was solvated in a large TIP3P (Jorgensen et al. 1983) water box with initial dimensions: 62 Å $\times$ 66 Å $\times$ 61 Å and comprised of 6133 water molecules, 17 Na$^+$ ions and 17 Cl$^-$ ions. This set-up models an isolated glycan, separated from its periodic images by approximately 60 Å, vertical to the imaginary surface spanned by the *xz* plane of the simulation box, with its asparagine end at the base (see schematic representation in Fig. 2). All MD simulations were performed with NAMD 2.9 (Phillips et al. 2005). The oligosaccharide sequence was generated according to the all-atom GLYCAM06h (Kirschner et al. 2007) parameter set for oligosaccharides and glycoproteins using the website interface (Woods Group 2005).

During the simulation, a constant temperature of 300 K was maintained by weak coupling to an external bath, using the Berendsen thermostat (Berendsen et al. 1984). Use of this particular thermostat was essential in combination with flow, because of the particular algorithm used for scaling. Using this method, the temperature of the system is corrected such that the deviation from the assigned temperature exponentially decays with specified constant $\tau$ (In this case $\tau = 0.2$ ps$^{-1}$). Because the re-scaling happens for each coupled atom irrespectively of its velocity, the Berendsen thermostat allows for the preservation of low/high as well as directionally biased regions of flow. By contrast, a thermostat that rescales the velocities by adding random noise from a normal distribution would smooth out the effect of flow.

A constant pressure of 1 atm was maintained with isotropic position scaling and a pressure relaxation time of 100 fs. All bond lengths for hydrogens were constrained to their equilibrium values through application of the SHAKE algorithm (Ryckaert et al. 1977). The dynamics simulations were not in any way constrained to reproduce experimental observables.

The cut-off distance, common for electrostatic and van der Waals calculations, was set to 12.0 Å. All pairs connected directly through a third atom were excluded from non-bonded calculations, whereas pairs connected directly through two intermediate atoms were not scaled, in accordance with the force field documentation (GLYCAM06). The non-bonded interactions pair list was generated for distances over 13.5 Å.

The timestep was set to 2 fs. Short-range non-bonded interactions were calculated in every timestep. Full electrostatic calculations were performed every two timesteps, and the atom list reassignment cycle was ten timesteps. Particle Mesh Ewald (Essmann et al. 1995) was employed for long-range electrostatic interactions.

Parameters were identical for both diffusion and flow simulations. The water flow was implemented as an additional force in the direction of the *x*-axis applied to the oxygen water atoms. This method is similar to the one used by Koplik et al. (1988, 1989) and later by Sokhan et al. (2002) and Kotsalis et al. (2004). The value of the force for the simulation results presented here was $5 \times 10^{-4}$ kcal/mol Å (approx. $0.35 \times 10^{-2}$ pN per atom), and it was applied in every timestep (2 fs).

The choice of the accelerating force is a trade-off between too small (and therefore the thermostat completely counter-balances the flow) and too large where the bulk velocity becomes comparable to diffusion atomistic velocity of the water particles as the presence of the glycan fails to slow down the water acceleration. Finding an appropriate force magnitude was achieved by trial and error and will always depend on the specific system under study.

In the absence of the tethered glycan, the flow would accelerate indefinitely. In order to simulate the wall shear stress (WSS) effect of the flow, the glycan was tethered to its equilibration coordinates by its asparagine end, using harmonic Cartesian restraints (fixed atoms: C, C$_{\upalpha }$, C$_{\upbeta }$, C$_{\upgamma }$, N) with a restraint barrier of 10 kcal/mol A$^2$.

Coordinates and velocities were saved every 0.4 ps, giving 500,000 data frames of both flow and diffusion trajectories to be analysed by the ptraj module of the Amber molecular dynamics package (Case et al. 2012). Two-dimensional torsion angle distribution heat maps were generated using Boltzmann population weighting (Landau and Lifshitz 1975). Images of molecular structure were generated using the molecular visualisation program VMD (Humphrey et al. 1996). The simulation real time for 200 ns of diffusion simulations using 64 cores was 11 days. Using identical computational resources, flow simulations were three times more expensive at 33 days for 200 ns.

## Results and discussion

**Fig. 3:**
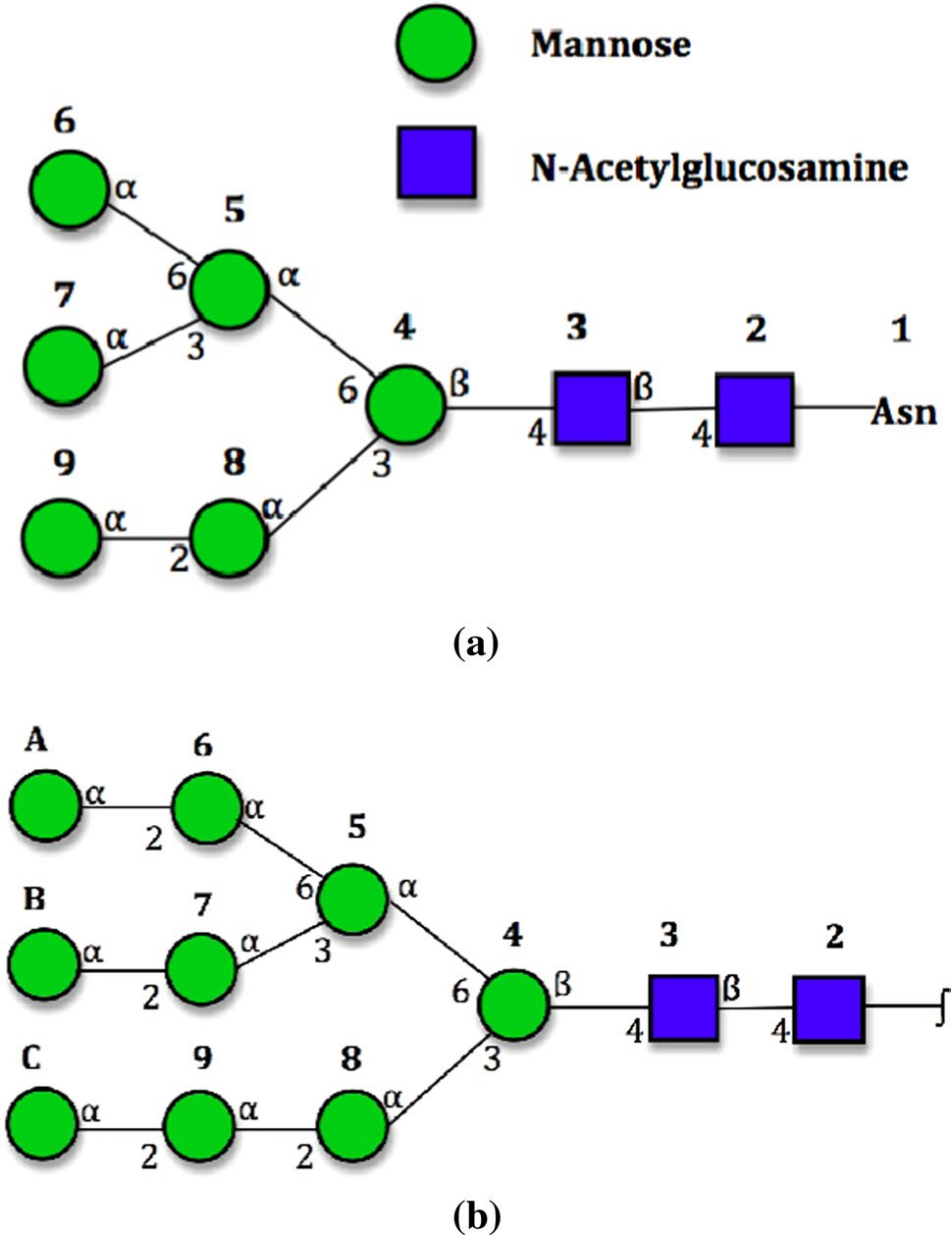
Side-by-side comparison of the six and nine mannose glycans. **a** $Man_6GlcN\!Ac_2$ used in the present work and **b** $Man_9GlcN\!Ac_2$ in NMR experimental study (Woods et al. 1998). Residues A, B and C are absent in the glycan used for the present computational model

We have carried out two conformational analyses of $Man_6GlcNAc_2$, using unrestrained (with the exception of Cartesian restraints on the aglycon residue) molecular dynamics calculations, in diffusion as well as water flow conditions. Figure S1 in supplemental material contains snapshots of the simulation during the second half of the evolution of the system (100–200 ns in 20 ns intervals). As part of the analysis, the molecular dynamics trajectories were used to back-calculate certain NMR parameters, such as inter-proton nuclear Overhauser effects (NOEs) (see Supplemental Material Section 3 for details on the methodology). The findings were further compared with results from experimental NMR measurements of $Man_9GlcNAc_2$ (Woods et al. 1998) (a schematic comparison between the two glycans is shown in Fig. 3).

Investigation of ion hydration properties as well as ion–ion interactions via calculation of radial distribution functions, for the case of diffusion, yielded results identical to those of Eriksson et al. as reported, using the GLYCAM04 (Woods et al. 1995) and AMBER ff99 (Wang et al. 2000) force fields and is included as supplemental material (Figs. S.2, S.3). We also looked at the effect of flow in the ion distribution about the solute, resulting from the biasing force of the water on the ions themselves as well as the reorientation of the glycan. Per sugar-residue radial distribution calculations revealed no major differences between static and flow conditions.

### Calculation of imposed flow

The water bulk velocity in the direction of the *x*-axis, as imposed by the added force, was calculated by averaging over instantaneous velocities in the trajectory.

1 $$\begin{aligned} v_{x(\text{bulk})} = \frac{\sum \nolimits _{j=1}^{m} \sum \nolimits _{i=1}^{n} \frac{v_{xij}}{n} }{m}, \end{aligned}$$

where $n = 6133$, the number of water molecules, $m= 500{,}000$, the number of frames and $v_{xi}$ the instantaneous velocity of water molecule *i* in the timestep at which the frame is saved.

According to Eq. (1), the water bulk velocity on the *x*-axis for all reported results was $v_{x(\text{bulk})} = 0.50 \pm 0.15$ m/s, for the added force discussed.

We believe that the flow rate, although still on the upper limit of what is expected of near-wall velocity even in large vessels (Bammer et al. 2007), is more relevant to the problem than rates reached in similar studies, where bulk velocities of 10–50 m/s are not uncommon (Chen et al. 2008; Zou et al. 2010; Cruz-Chu et al. 2014). In larger arteries which can be of several centimetres in diameter, mean blood velocities reach 0.2 m/s and peak velocities can go as high as 1 m/s. As a reference, the mean middle cerebral artery blood velocity has a mean value of 0.52 m/s as reported in studies on athletes (Nybo and Nielsen 2001) at rest. However, it is recognised that near-wall velocities are expected to be considerably lower.

The flow imposed is considered continuous rather than pulsatile, given the timescale of the MD simulation which is small compared to typical oscillatory flow frequencies. However, the imposed flow velocity is indeed oscillatory, however, at a much higher frequency than pulsatile blood velocities.

In order to investigate the relationship between the force imposed per oxygen atom and the resulting flow, simulations of the exact same system were performed for the same number of timesteps but with 10% less force imposed. As this is not a straightforward process (many forces operate on each individual atoms, including the effect of the thermostat), the resulting flow was not proportionally low. In fact, the calculated slower flow was 60% of the original: $v_{x(bulk_slow)} = 0.30 \pm 0.14$ m/s.

The magnitude of the force used to drive the flow was calculated using trial and error, allowing the system to run for a few nanoseconds at a time to estimate the average velocity generated. The method used to impose flow is computationally expensive and therefore does not allow for long trial and error simulations, which makes it difficult to accurately estimate the resulting velocities. This is the reason why only two different regimes were compared in full.

### Effect of the tethered glycan on the water velocity profile

**Fig. 4:**
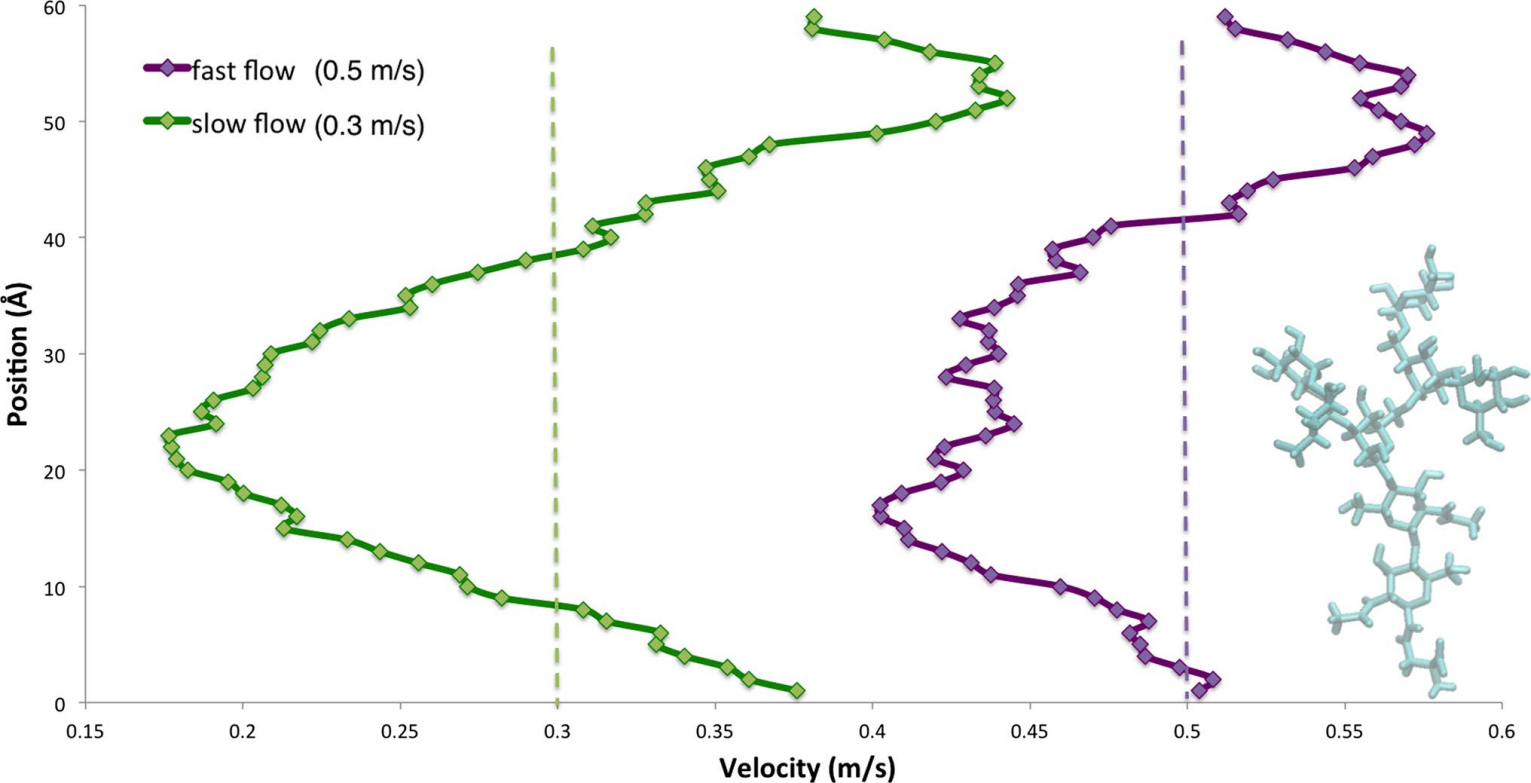
Velocity profiles across the *y*-axis. The dotted lines mark the average velocity for the normal (green) and reduced (purple) flow. Solid lines indicate average velocity for that segment of the *y*-axis, and the embedded schematic of the stretched conformation is to scale. The schematic denotes the highest possible level the constrained glycan can reach. Due to periodicity, this velocity profile is repeated above and below the end-points of the *y*-axis (there is no boundary effect). The water velocity profile across the *y*-axis was calculated by averaging the individual water oxygen velocities $v_x$ of overlapping 7 Å-wide slabs of the simulation box. In the case of the larger driving force, the average velocity seems smaller than the one indicated by the dashed line. This is an artefact of the per-slab velocity calculation, as the layers in the slabs overlap. The dotted line therefore indicates the true mean velocity as calculated for all water molecules (colour figure online)

The water velocity profile across the *y*-axis was calculated by averaging the individual water oxygen velocities $v_x$ of overlapping 7 Å-wide slabs of the simulation box (Fig. 4). Despite the flexibility of the molecule and heat motion of the water which generates a significant amount of noise, it can be observed that, as expected, along the length of the glycan’s range of movement, the velocity begins to drop, particularly as the shape of the molecule becomes increasingly dendritic, and in accordance with the most populated configurations that we have discussed. The glycan is able to stretch to a maximum of 33 Å  after which the velocity begins to rise again and, at the furthest point from the glycan, it reaches its peak. Such a depiction encapsulates several mechanisms influencing the impact of the oligosaccharide moiety on the flow: effect of highly populated conformations, maximum glycan extension, and coupled molecule–water motion are simultaneously captured and result in the velocity distribution shown in the figure.

Although the velocity profile is similar for both flow regimes, it can be observed that the reduced flow regime produces a higher drop within the glycan’s range of movement in comparison with the faster flow. This indicates that by further reducing the force which drives the flow, very quickly the velocity on the *x*-axis could become unsustainable, as the force will not be enough to compensate for the “friction” generated by the non-bonded interactions of the glycan and water and the equilibrating effect of the thermostat.

### Conformational analysis and flow-induced orientation changes on the glycan

**Fig. 5:**
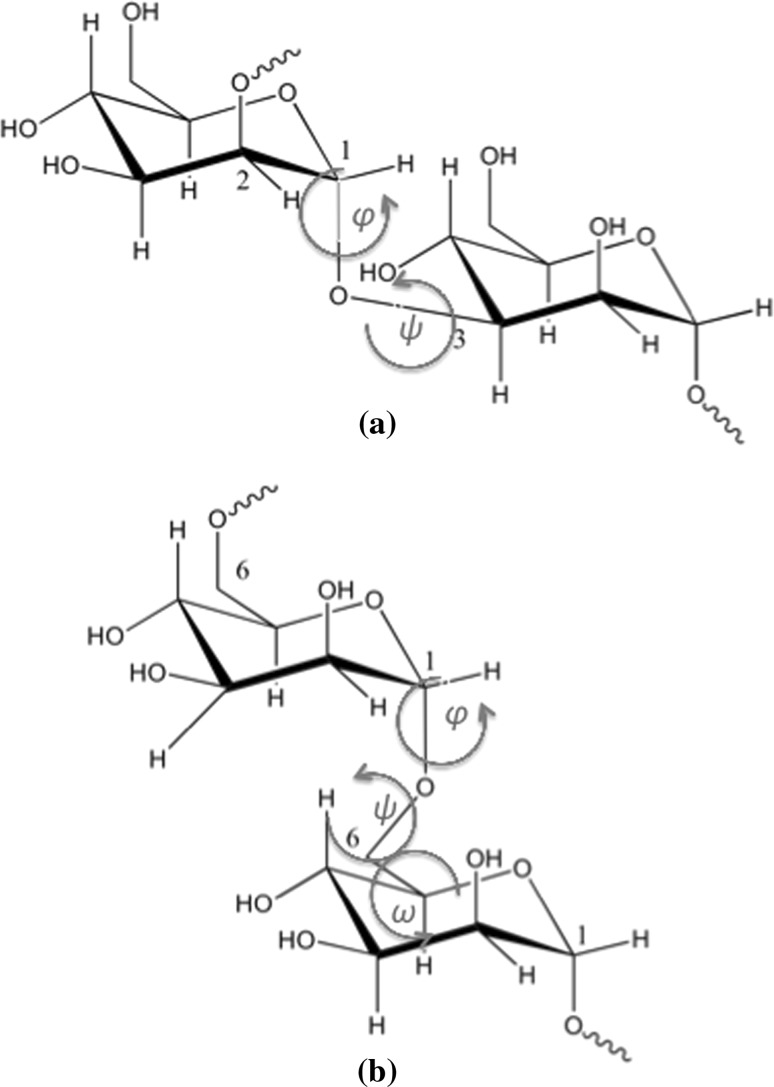
Detailed schematic of glycosidic linkages and associated dihedral angles. **a** 1,3 linkage between residues 8 and 4 ( $\phi$ and $\psi$) and **b** 1,6 linkage between residues 5 and 4 ($\phi$, $\psi$ and $\omega$)

**Fig. 6:**
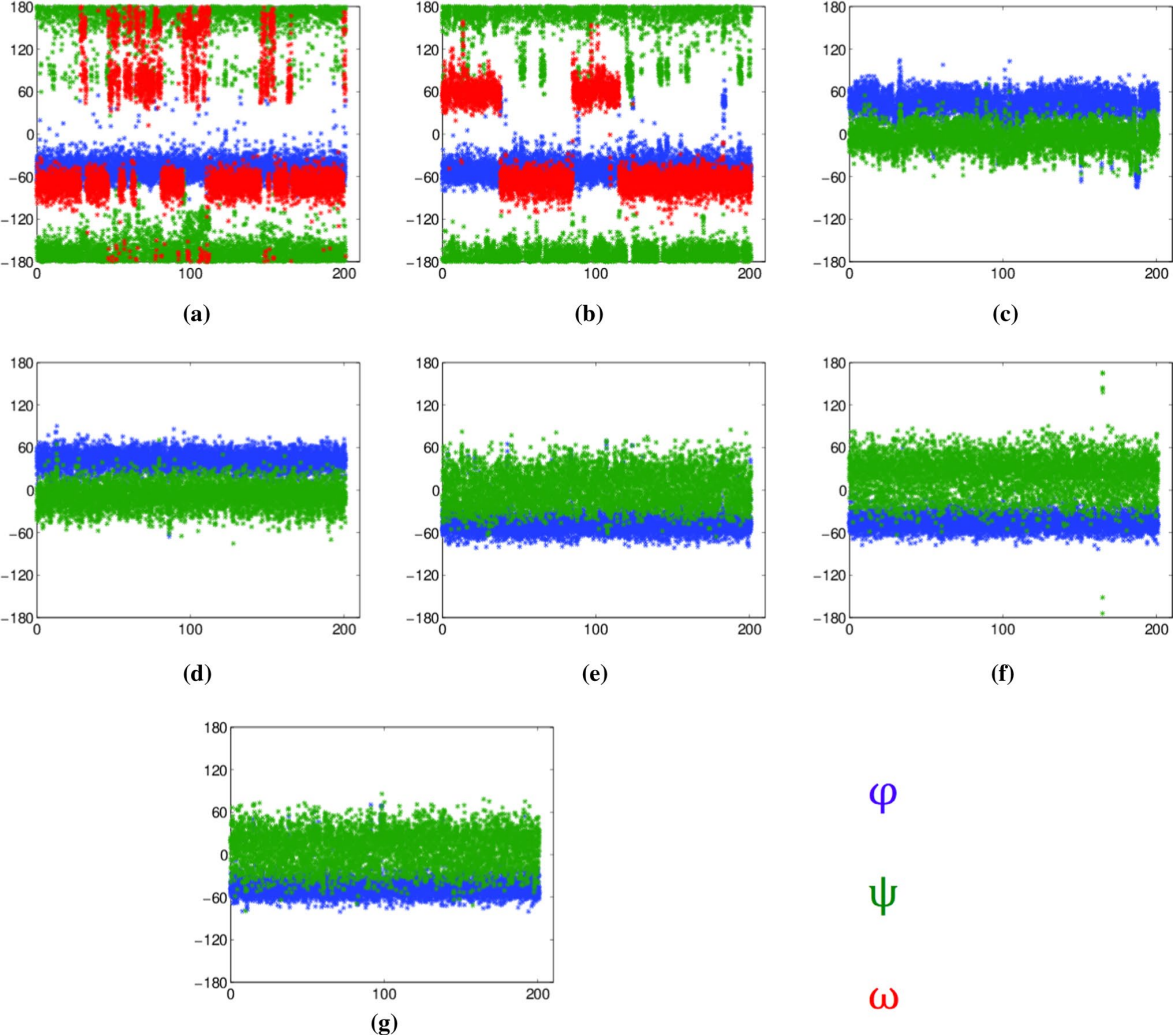
Evolution of torsion angles during the simulation for all glycosidic linkages **a** 6-5, **b** 5-4, **c** 4-3, **d** 3-2, **e** 7-5, **f** 9-8 and **g** 8-4 in $Man_6GlcNAc_2$. Colours used in all graphs: blue for $\phi$, green for $\psi$ and red for $\omega$. The data were obtained from 200 ns of unrestrained MD simulations in water (colour figure online)

Figure 5 contains a schematic representation of two different kinds of linkages, namely the 1,3 linkage between residues 8 and 4 and the 1,6 linkage between residues 5 and 4. In Fig. 6, we present plots of torsion angles versus simulation time of 200 ns for all glycosidic linkages in $Man_6GlcN\!Ac_2$. They are essential in extracting information on the adequacy of sampling of conformational states for the glycan in solution. All plots correspond to the diffusion trajectory torsion angles calculated using the following definition: for $(1-x)$ linkages, where $x = \{1,2,3,4\}$: $\phi = \text{H}_1{-}\text{C}_1{-}\text{O}{-}\text{C}_x^{\prime }$ and $\psi = \text{C}_1{-}\text{O}{-}\text{C}_x^{\prime }{-}\text{H}_x^{\prime }$, and for the (1-6) linkages: $\phi = \text{H}_1{-}\text{C}_1{-}\text{O}{-}\text{C}_6^{\prime }$, $\psi = \text{C}_1{-}\text{O}{-}\text{C}_6^{\prime }{-}\text{C}_5^{\prime }$ and $\omega = \text{O}{-}\text{C}_6^{\prime }{-}\text{C}_5^{\prime }{-}\text{O}_5^{\prime }$.

Corresponding results for flow do not significantly differ (see Supplemental Material, Fig. S.4), with a few exceptions that will be discussed later in this section. The 1, 2 and 1, 3 linkages (Fig. 6e–g) principally occupied the $-\,60^\circ \phi$ angle and $0^\circ \psi$. The 1,4 linkages (Fig. 6c, d) remained at $+\,60^\circ \phi$ and $0^\circ \psi$ throughout the simulations.

**Fig. 7:**
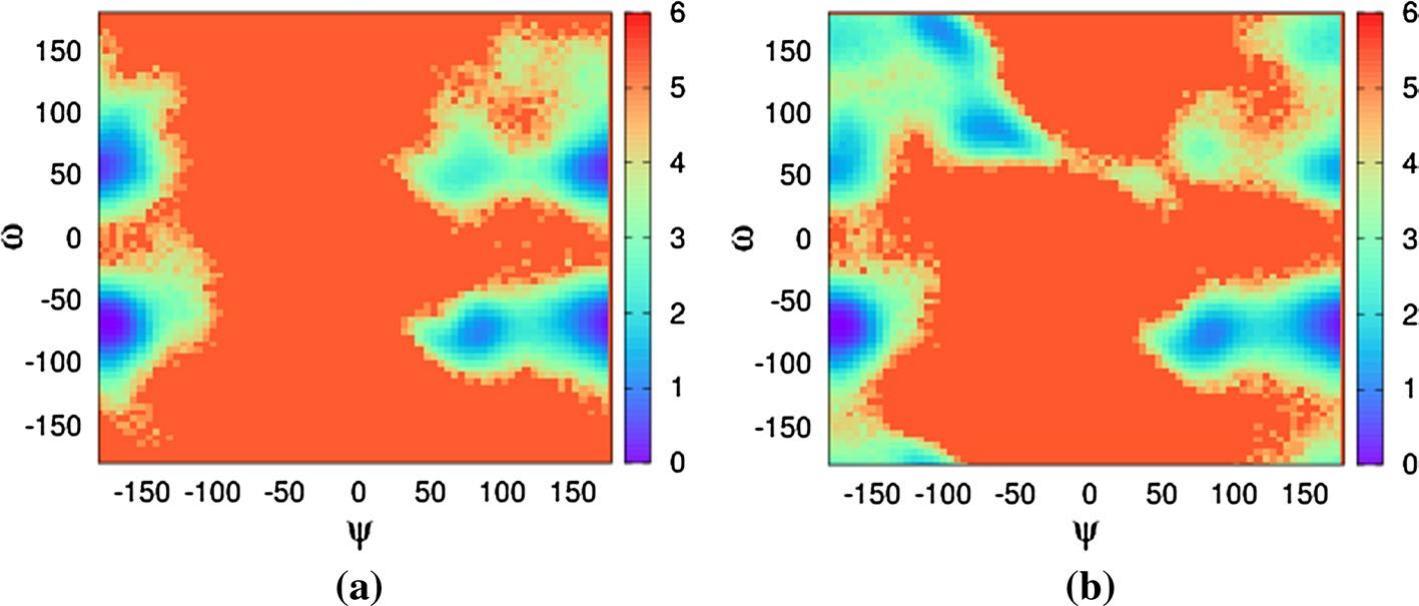
Boltzmann energy heat maps in the 5-4 linkage. Population occupancy of torsion angle ${\varvec{\psi }}$ versus torsion angle ${\varvec{\omega }}$. **a** In diffusion and **b** in flow. Energy colour bar in kcal (colour figure online)

The more flexible 1,6-glycosidic linkages (Fig. 6a, b) show one state for $\phi$, at $-\,60^\circ$, and two states for $\psi$, mostly $180^\circ$ with some $60^\circ$. The $\omega$ angle in both linkages shows that the $-\,60/+\,60^\circ$ states are dominant though 6-5 is a mixture of $180^\circ$, $60^\circ$ and $-\,60^\circ$ while 5-4 only has $-\,60/+\,60^\circ$. Interestingly, the only difference between diffusion and flow comes from the 5-4 linkage, specifically the $\psi$ torsion angle (Fig. 7), which occupies a new $-\,60^\circ$ state, not explored at all in diffusion. This state is also not observed in the slower version of the flow. One possible explanation is that this new state, exclusive to the faster flow, has a low occurrence frequency and that the flow provides the necessary energy for more frequent transitions.

Table 1 contains the calculated and experimentally observed NOEs for the asparagine-linked $Man_6GlcN\!Ac_2$ and $Man_9GlcN\!Ac_2$, respectively. It is important to highlight that, although $Man_6GlcN\!Ac_2$ and $Man_9GlcN\!Ac_2$ are similar, the presence of a terminating residue on each of the three branches could alter the branching linkage conformations. Nonetheless, the NOE distance values calculated from the diffusion trajectory are in good agreement with experimentally derived ones. Discrepancies of about $1.0 \AA$ are observed in the $\alpha 1 \rightarrow 2 \text{H}_1/\text{H}_3^{\prime }$ distance between residues 9 and 8, the $\alpha 1 \rightarrow 3 \text{H}_1/\text{H}_1^{\prime }$ and $\text{H}_1/\text{H}_4^{\prime }$ distances between residues 8 and 4 and the $\alpha 1 \rightarrow 3 \text{H}_1/\text{H}_4^{\prime }$ distance between residues 7 and 5. In all cases, the NOE distances appear increased, potentially due to insufficient sampling, that is, the possibility that conformations with short proton–proton distances were either not sampled or under-sampled. We show in the following analysis performed on the 8-4 and 9-8 linkages that the latter is not the case.

**Table 1:** Calculated inter-proton inter-residue distances in ${Man_6GlcN\!Ac_2}$ compared to experimental observations in ${Man_9GlcN\!Ac_2}$ (Woods et al. 1998)

Linkage	Proton pair	Distance NMR (Å)	Distance MD diffusion (Å)	Distance MD flow (Å)
$$(\alpha 1 \rightarrow 2)$$				
9-8	$\text{H}_1/\text{H}_1^{\prime }$	Obscured	3.19	3.20
	$\text{H}_1/\text{H}_2^{\prime }$	(2.10)	2.28	2.28
	$\text{H}_1/\text{H}_3^{\prime }$	3.23	4.28	4.29
	$\text{H}_1/\text{H}_4^{\prime }$	No-NOE	4.40	4.43
	$\text{H}_5/\text{H}_1^{\prime }$	(2.74)	2.45	2.43
$$(\alpha 1 \rightarrow 3)$$				
8-4	$\text{H}_1/\text{H}_1^{\prime }$	3.30	4.62	4.62
	$\text{H}_1/\text{H}_2^{\prime }$	3.11	3.41	3.40
	$\text{H}_1/\text{H}_3^{\prime }$	Short	2.27	2.27
	$\text{H}_1/\text{H}_4^{\prime }$	2.98	3.98	3.96
	$\text{H}_5/\text{H}_1^{\prime }$	No-NOE	4.88	4.86
	$\text{H}_5/\text{H}_2^{\prime }$	Obscured	2.53	2.54
7-5	$\text{H}_1/\text{H}_1^{\prime }$	No-NOE	No-NOE	No-NOE
	$\text{H}_1/\text{H}_2^{\prime }$	2.99	3.54	3.47
	$\text{H}_1/\text{H}_3^{\prime }$	2.09	2.30	2.29
	$\text{H}_1/\text{H}_4^{\prime }$	2.90	3.89	3.97
	$\text{H}_5/\text{H}_1^{\prime }$	No-NOE	4.95	4.90
	$\text{H}_5/\text{H}_2^{\prime }$	2.69	2.51	2.50
$$(\alpha 1 \rightarrow 6)$$				
5-4	$\text{H}_1/\text{H}_6^{\prime }$	2.75	2.86	2.66
	$\text{H}_1/\text{H}_6^{\prime }$	2.41	2.42	2.47
	$\text{H}_1/\text{H}_5^{\prime }$	3.27	3.57	4.01
6-5	$\text{H}_1/\text{H}_6^{\prime }$	(2.40)	2.75	2.78
	$\text{H}_1/\text{H}_6^{\prime }$	2.14	2.44	2.43
	$\text{H}_1/\text{H}_5^{\prime }$	Obscured	4.03	3.70

Distances derived from overlapping peaks are indicated in parentheses. Where no-NOE is mentioned, distances are usually ${>\,4.0}$ Å

**Fig. 8:**
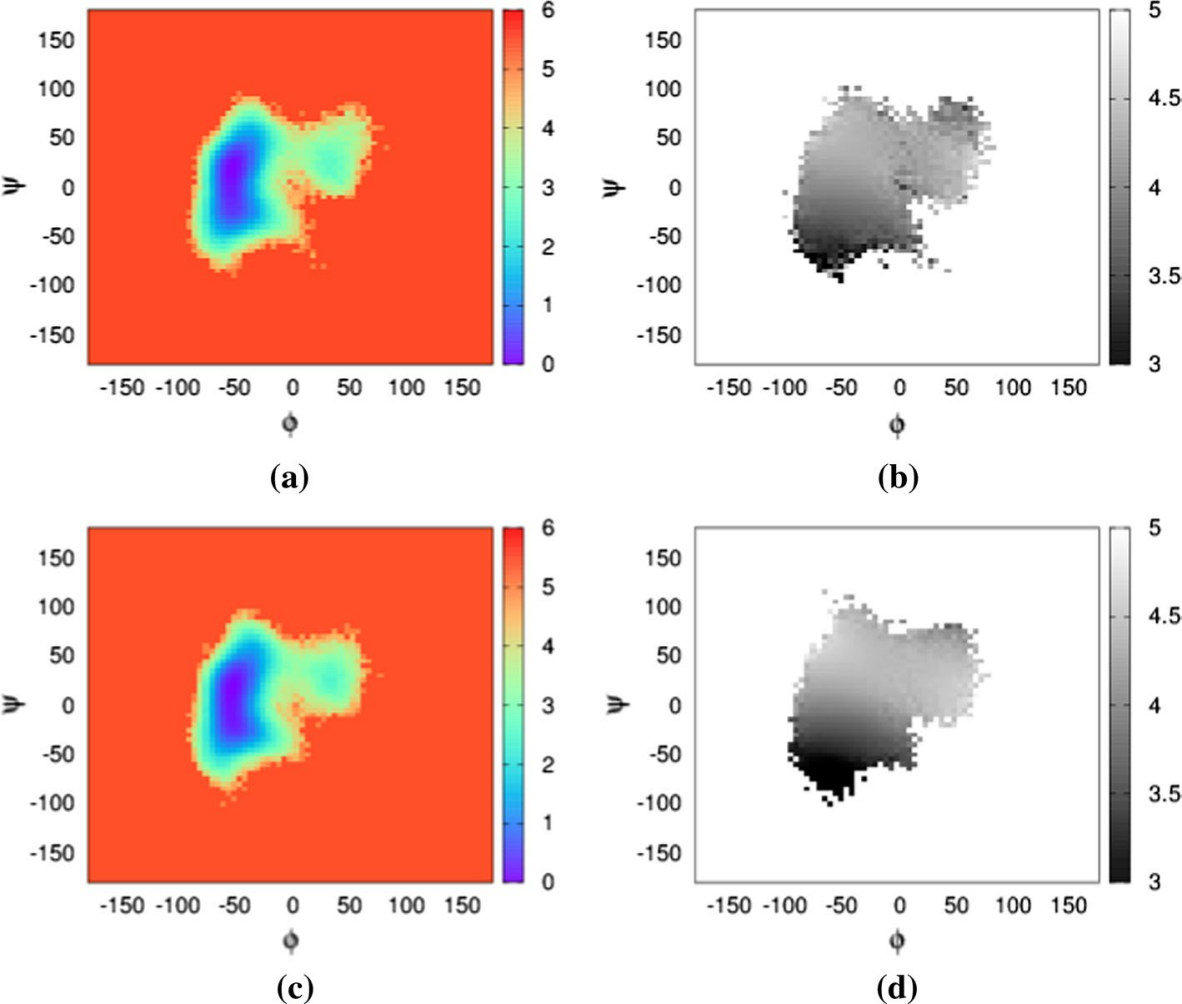
Distance and Boltzmann energy heat maps for the 8-4 linkages. Ramachandran plot of $\phi$ versus $\psi$ angles, colour-coded according to Boltzmann energy distribution for **a** in static conditions, **c** in flow conditions. Linkage Ramachandran plot of $\phi$ versus $\psi$ angles, grey-scaled according to distance between protons $\text{H}_1$ and $\text{H}_4^{\prime }$ for **b** static conditions and 800 flow conditions. All colour bars in kcal, greyscale bar in Å. **a** 8-4 linkage—static. **b** 8-4 linkage—static. **c** 8-4 linkage—flow. **d** 8-4 linkage—flow (colour figure online)

**Fig. 9:**
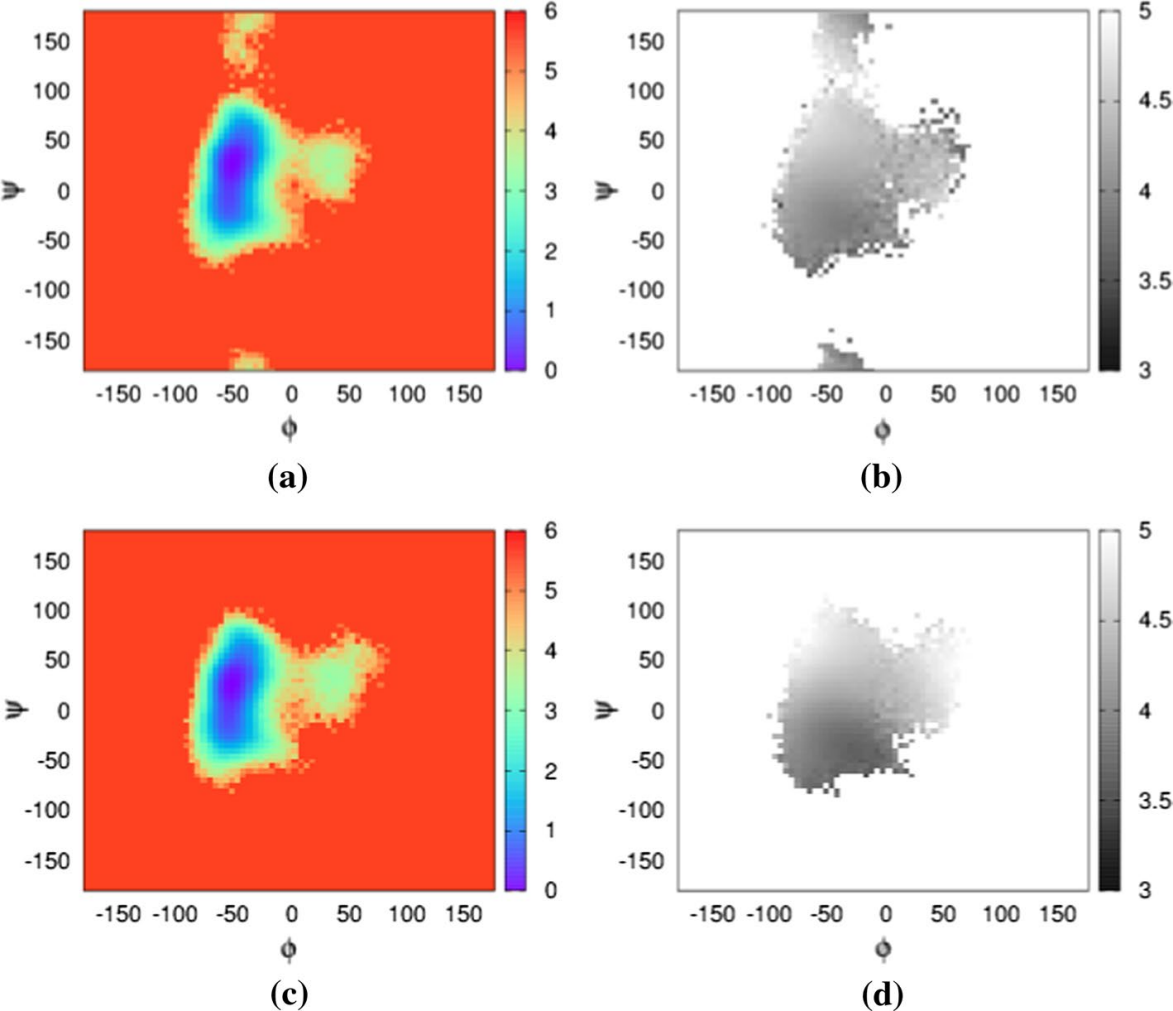
Distance and Boltzmann energy heat maps for the 9-8 linkages. Ramachandran plot of $\phi$ versus $\psi$ angles, colour-coded according to Boltzmann energy distribution for **a** in static conditions, **c** in flow conditions. Linkage Ramachandran plot of $\phi$ versus $\psi$ angles, grey-scaled according to distance between protons $\text{H}_1$ and $\text{H}_4^{\prime }$ for **b** static conditions and **d** flow conditions. All colour bars in kcal, greyscale bar in Å. **a** 9-8 linkage—static. **b** 9-8 linkage—static. **c** 9-8 linkage—flow. **d** 9-8 linkage—flow (colour figure online)

In the case of the 8-4 glycosidic linkage, for which analysis of the simulation trajectory results in short NOE distance in comparison with experimental values given in Table 1, only one conformation has been sampled (Fig. 6g). The Boltzmann energy distribution of the $\phi$ versus $\psi$ angle in diffusion (Fig. 8a), when examined together with the average $\text{H}_1 - \text{H}_4^{\prime }$ proton distance map (Fig. 8b), indicates no sampling bias towards long distances. This is also true in the case of flow. The behaviour of the 9-8 glycosidic linkage is similar when examining the average $\text{H}_1 - \text{H}_3^{\prime }$ proton distance map (Fig. 9b) alongside the Boltzmann energy distribution in diffusion (Fig. 9a).

The NOE distance values calculated from the flow trajectory did not present statistically significant differences in comparison with diffusion, as both sets of values qualitatively describe the same molecular structure. Furthermore, there were no significant differences between this case and the “slow” flow regime, which is why relevant results are not included in the table.

Given the above analysis, and assuming that corresponding NOE data between $Man_6GlcN\!Ac_2$ and $Man_9GlcN\!Ac_2$ should match, we can dismiss the explanation of under-sampling a population of conformations. Also, the assumption that the tumbling of the glycan has no effect on the NOE calculation may also result in some variation, in agreement with experiment. This is explored in a recent study on sucrose published by Chalmers et al. (2016), where they argue that, for long-range NOEs in flexible molecules, long-range distances will be off due to the internal motion of the glycan. The method they propose takes these conformational changes into account as well as the distances, and although it is certainly more correct, it does requires exceptionally long simulations that would have been unfeasible for the purposes of the current work.

However, it is also possible that short-distance conformations were not at all sampled in the MD experiment. Given the timescale of NMR (ms) in comparison with MD (ns) and the fact that we did not constrain the starting conformation based on the NMR experiment, this is, indeed, a plausible explanation.

The remaining Ramachandran plots of $\phi$ versus $\psi$ angles, colour-coded according to Boltzmann energy distribution for the 3-2, 4-3, 5-4, 6-5 and 7-5 linkages in diffusion and flow as well as plots of $\psi$ versus $\omega$ angles for the 6-5 linkage, can be found in the supplemental material (Figs. S.5, S.6).

### Effect of flow on the amino acid

So far in our analysis, results point towards minor or insignificant changes in the glycan conformation, as characterised by NOE distances and glycosidic linkage torsion angles. There is, however, an alteration on the orientation of the glycan, which is attributed to the flow. In this section, we investigate whether this information would translate to the transmembrane protein, which is relatively stiff and immobile, therefore unable to align itself with the flow in the same way the flexible glycan does.

**Fig. 10:**
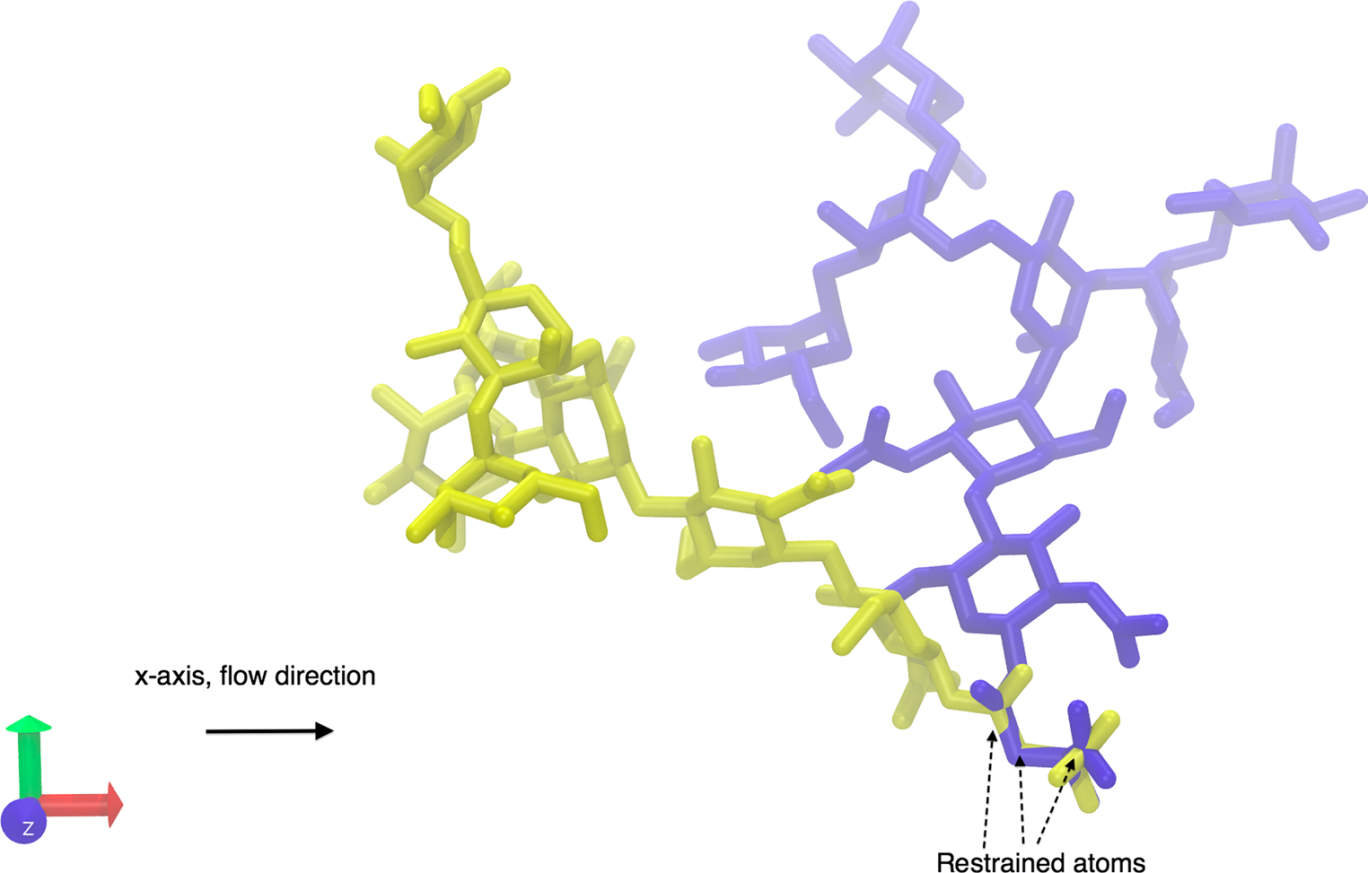
A representation of the two dominant states in diffusion and flow. The yellow and blue molecules represent the two distinct highly populated states favoured by the glycan. While both states are observed in flow and diffusion, the yellow molecule’s conformation was dominant in diffusion whereas the blue molecule conformation was dominant in flow conditions (colour figure online)

Two conformational states were identified, which varied between the flow and diffusion simulations. The conformation of these two states was entirely dependent on the orientation of the glycan relative to the amino acid aglycone, defined by the torsion angles according to the following atoms’ positions $\phi$: $\text{C}_{\upbeta }$, $\text{C}_{\upgamma }$, N, $\text{C}_1^{\prime }$, $\psi$: $\text{C}_{\upgamma }$, N, $\text{C}_1^{\prime }$, $\text{C}_2^{\prime }$ and $\chi _2$: $\text{C}_{\upalpha }$, $\text{C}_{\upbeta }$, $\text{C}_{\upgamma }$, N for the asparagine amino acid ($\text{C}_1^{\prime }$, $\text{C}_2^{\prime }$ correspond to residue number 2. $\text{C}_{\upalpha }$, $\text{C}_{\upbeta }$ and $\text{C}_{\upgamma }$ are all restrained atoms, whereas the remaining are not). The effect that this linkage has on the orientation of the glycan is shown in Fig. 10 using the average $\phi$, $\psi$ and $\chi _2$ angles for each of these two states.

**Fig. 11:**
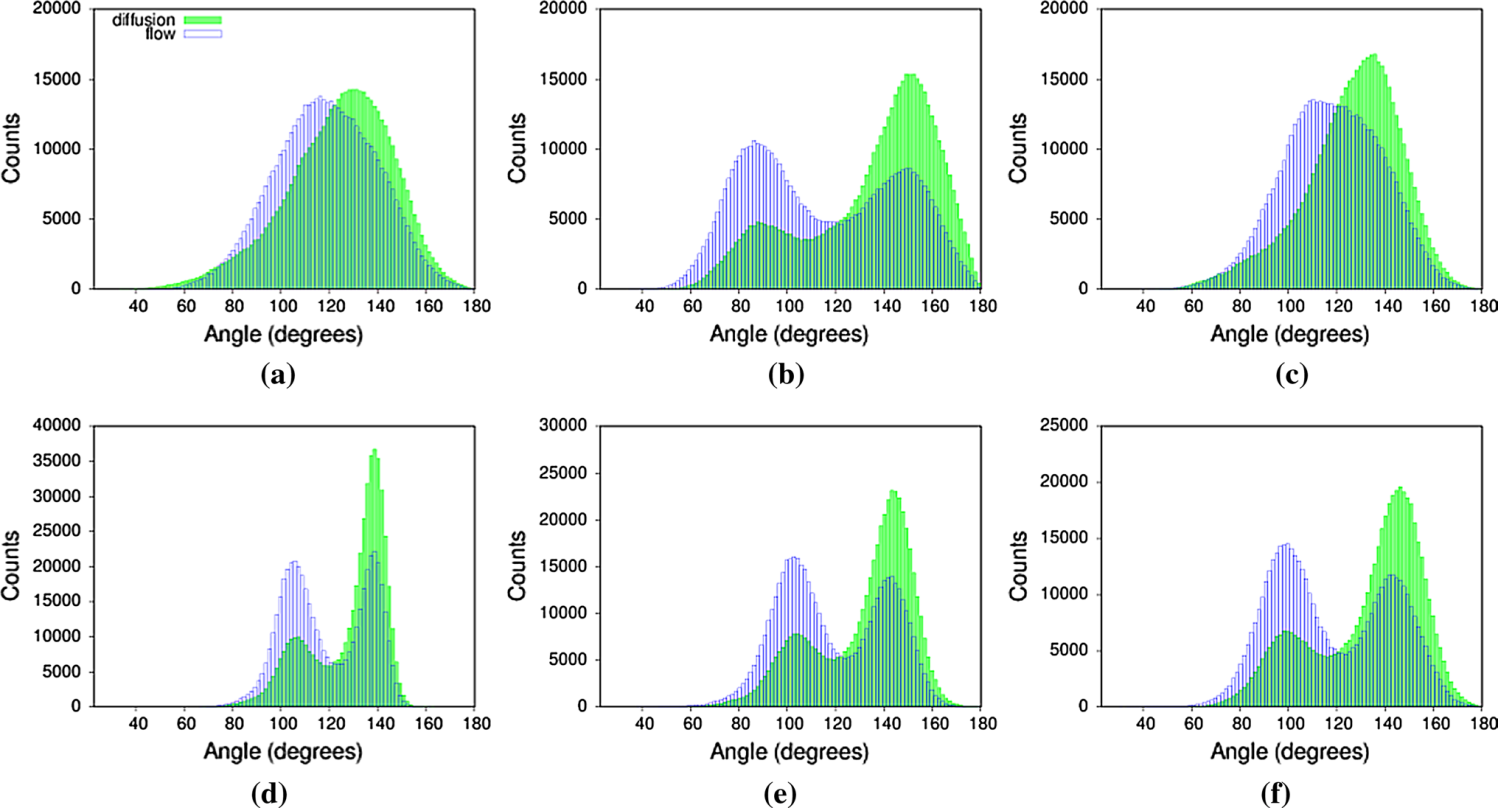
Calculated angles characterising the orientation and bending of the glycan. All angles are of the form $\angle ABx$, where A and B are the restrained atoms $\text{C}_{\upalpha }$ and $\text{C}_{\upbeta }$, respectively, and point *x* corresponds to the centre of mass of the residues **a** $\angle AB6$, **b** $\angle AB9$, **c** $\angle AB5$, **d** $\angle AB2$, **e** $\angle AB3$ and **f** $\angle AB4$

In order to quantify the orientational changes flow introduces to our system, we have compared characteristic angles formed between two fixed points on the asparagine end and the individual branches of the glycan in the diffusion and flow trajectories (Fig. 11). All angles are of the form $\angle ABx$, where *A* and *B* are the restrained atoms $\text{C}_{\upalpha }$ and $\text{C}_{\upbeta }$, respectively, and x is the centre of mass of the residue. In both flow and diffusion, the sugar residue appears in two principal states, as evidenced by the peaks on Fig. 11b, d–f.

**Fig. 12:**
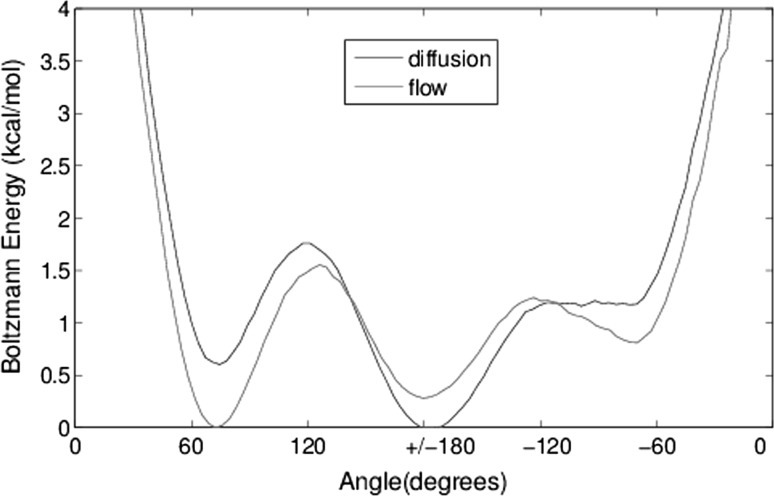
Boltzmann energy plots of the $\chi _2$ angle populations for the three aglycon (asparagine) conformational states. The energy shift between diffusion and flow is pronounced especially for the $+\,60$ and $\pm \,180$ angles

Flow affects the angle distribution in two ways: By inverting the principal state populations, making smaller angle values the dominant state, as seen for residues 2, 3, 5 and 9, which represent one branch of the glycan (Fig. 11b, d, e, f) and also by shifting the population towards the lower angle values in the case of residues 5 and 6, on the other branch (Fig. 11a, c), and this dominance switches between the two states. This transition favours the orientation of the glycan, which is most closely aligned with the flow vector as demonstrated in Fig. 10.

To illustrate the effect of flow on the tethered asparagine end of the glycan, we plot Boltzmann energy $\chi _2$ angle populations (Fig. 12). Of the three distinct highly populated states, the $\chi _2$ value of $\pm \,180^\circ$ is dominant in diffusion, a result consistent with crystallographic experiments (Petrescu et al. 2004), whereas in flow, the dominant state shifts to the lower $\chi _2$ values around 60$^\circ$. The overall shift in energy is in the order of 1 kcal (+ 0.6 kcal for 60 over 180$^\circ$ in diffusion and − 0.3 kcal in flow). There is a third state in the − 60$^\circ$ which is not significantly populated in either case. For the case of reduced flow, the increased energy around 60$^\circ$ is still observed; however, the relative energy at $\pm \,180^\circ$ remains the same.

## Conclusion

The computational experiments described above are based on a model of a flexible glycocalyx molecule reorienting itself as a result of the flow, exerting a pulling force on the rigid transmembrane protein. This model does not involve the complexity of multiple strands of long, negatively charged glycosaminoglycans and therefore explores no aspect of their interaction in the electrolyte solution. Although the flow regime is the most realistic, in terms of magnitude, achieved in MD simulations of the glycocalyx to date, the solute velocities produced by the water oxygen acceleration method remain high with regard to the magnitude expected near the vessel wall. Furthermore, the simulation box was not large enough to study the difference in flow velocity directly above (and below, due to periodic boundary conditions) the glycan.

There are further challenges with regard to the flow implementation method. Firstly, it is hard to calibrate and requires a trial and error approach with results that are not reusable in different (e.g. larger) systems. Secondly, this technique has severe limitations for scaling up, besides the well-known issues of using large water boxes with explicit solvent that already increase simulation time dramatically even in the absence of flow.

Despite the model’s limitations, we can look at this simple glycan in order to assess the type and magnitude of the effects of flow we can expect on such flexible biomolecules.

Conformational analysis showed that, in the short term, flow does not significantly alter the conformation space explored by the glycan, as indicated by glycosidic torsion angles and proton distance calculations. In the case of one linkage exploring additional $\psi$ and $\omega$ angles, this could be a result of enhanced sampling the flow provides, rather than actual change in conformation. Our results are in good agreement with experimental values for both diffusion and flow conditions. Indeed, that is to be expected, given the relative magnitude of velocity attributed to flow in relation to Brownian motion.

As a result of tethering, the orientation of the glycan is limited to a specific range. Differences in tethering would produce a smaller or larger range of movement and could allow the glycan to align its orientation with the flow over time. This introduced “resistance” to alignment with flow gives rise to the difference in angle distribution, as measured from the asparagine end to the branches of the glycan. We have also shown that flow translates into a population shift for torsion angles in the asparagine end, in order to facilitate flow alignment. This change in the conformation properties of the aglycon could indicate that, in vivo, the long strands of polysaccharides exert a pulling force that changes the conformation of the transmembrane proteoglycans, essentially playing the part of the initial mechanical signal transmitted through to the cytoskeleton.

## Electronic supplementary material

Below is the link to the electronic supplementary material.

Supplementary material 1 (pdf 6142 KB)
