# Supplementary material for: Oligosaccharide model of the vascular endothelial glycocalyx in physiological flow
Source: Microfluid Nanofluidics. 2018 Jan 29;22(2):21. doi: 10.1007/s10404-018-2037-5 (PMC5847235; doi:10.1007/s10404-018-2037-5)
Supplement: Supplementary file 1 — Supplementary material 1 (pdf 6142 KB) [file 10404_2018_2037_MOESM1_ESM.pdf]

Microfluidics and Nanofluidics manuscript No.
(will be inserted by the editor)

Oligosaccharide Model of the Vascular Endothelial Glycocalyx in Physiological Flow

Supplemental Material

Maria Pikoula[1,2] · **Matthew B. Tessier**[3,4] ·
Robert J. Woods[5,6] · **Yiannis Ventikos**[7]

the date of receipt and acceptance should be inserted later

1 Trajectory

Figure 1 contains snapshots of the simulation during the second half of the evolution of the system (100-200ns in 20ns intervals). We can observe the glycan's range of motion which, due to the 5-atom restraint conditions imposed, can at most align itself perpendicular to the flow, but is never able to align itself to it. However, as discussed in the main text, the proportion of frames where the glycan is in aligned in favour to the flow is increased in comparison to diffusion (where the same constraints are also applied).

1. Department of Engineering Science, Institute of Biomedical Engineering, University of Oxford
OX1 3PJ, Oxford, United Kingdom

2. Farr Institute, UCL Institute of Health Informatics
222 Euston Road, London NW1 2DA , United Kingdom

3. Complex Carbohydrate Research Center, University of Georgia,
315 Riverbend Road, Athens, GA 30602, USA

4.Department of Chemistry, University of Georgia,
140 Cedar St, Athens, GA 30602, USA

5. Complex Carbohydrate Research Center, University of Georgia,
315 Riverbend Road, Athens, GA 30602, USA

6. School of Chemistry, National University of Ireland,
Galway, University Road, Galway, Ireland

7. Department of Mechanical Engineering, University College London
Torrington Place, London WC1E 7JE, United Kingdom
E-mail: y.ventikos@ucl.ac.uk

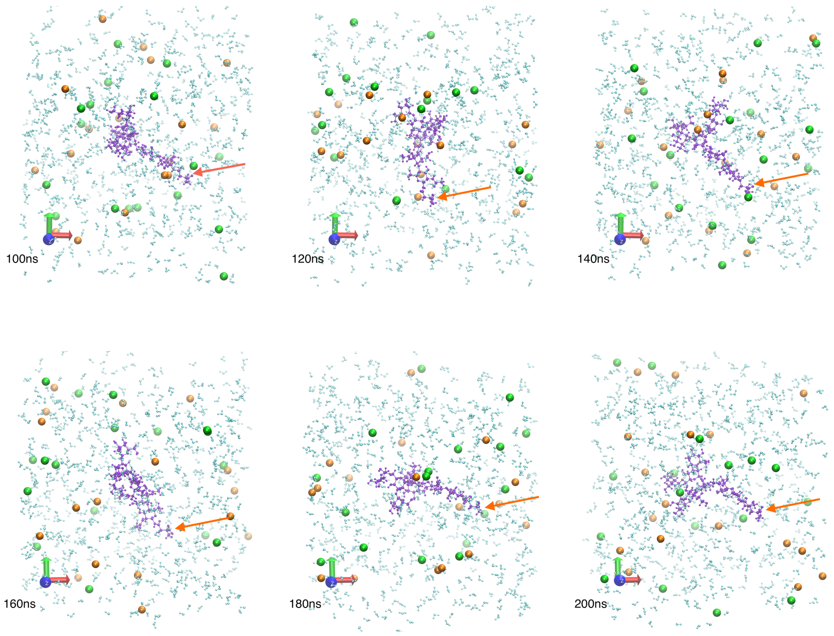

Fig. 1: **Snapshots of the flow trajectory centred around the $Man_6GlcNAc_2$ centre of mass** Showing the glycan (purple), Na^+ (orange) and Cl^- (green) ions as well as 1/20th of water molecules in the simulation box. The red arrow in the first panel indicates the tethering location on the aglycon. The axis in the bottom left corner indicates the x (red) y (green) and z (blue) direction. Flow is imposed parallel to the x axis.

2 Ion Hydration

Here we present ion hydration properties, corresponding to the ones investigated by Eriksson et al. [3] for the same system. They are characteristic of the simple diffusion system and useful for validation of the method used.

One of the main differences between Eriksson et al. and this work is the use of different force field parameters. Eriksson et al. used ion parameters from Beglov and Roux [1] whereas we have used parameters from the AMBER [6] [5] force field. They also performed reference simulations with ion parameters from the AMBER force field which resulted in a much higher tendency for ion-pairing in general. In particular, ion-pairing was enhanced approximately fivefold for K^+, as observed in the results of the present work (Figure 2).

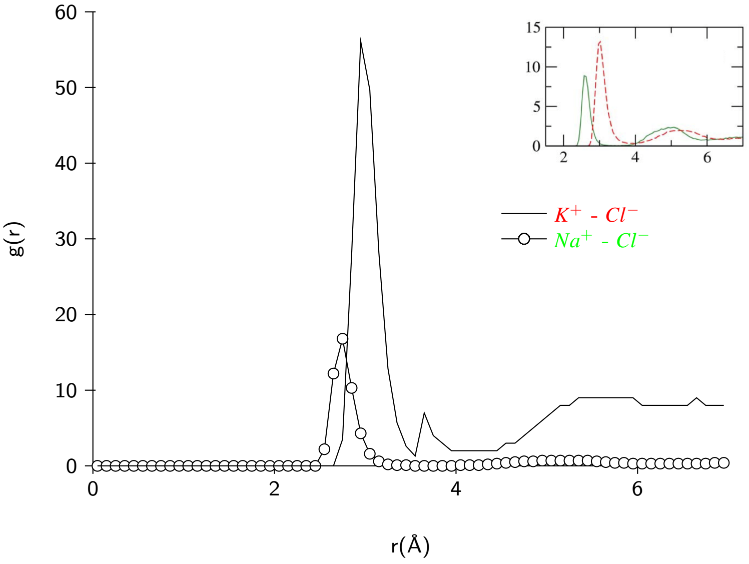

Fig. 2: Cl^- radial distribution functions of Na^+ and K^+.

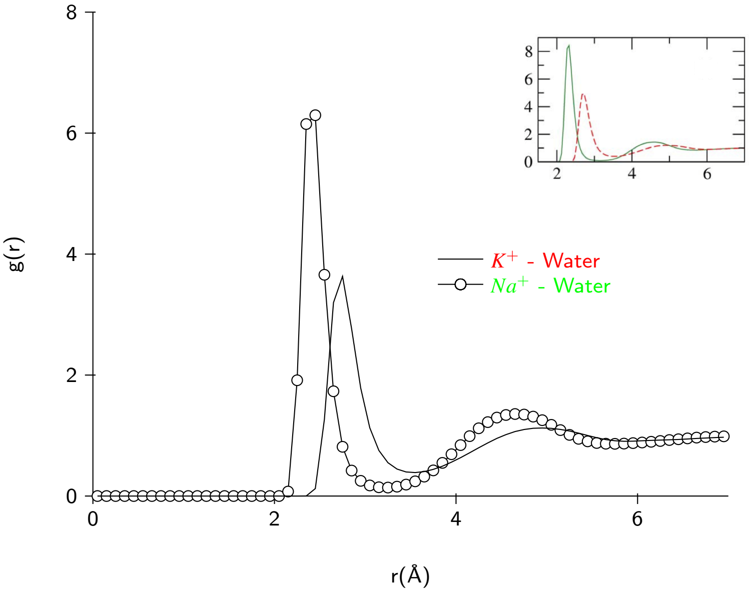

Fig. 3: Water-oxygen radial distribution functions of Na^+ and K^+.

3 Validation Methodology

Experimentally characterised 3-dimensional (3D) structures of carbohydrates are predominantly determined by fitting a set of model structures to nuclear magnetic resonance (NMR) observables [2] [4]. In typical NMR experiments, distances and torsion angles (angles formed by four atoms connected by three bonds in sequence) are determined from spectra of magnetically susceptible isotopes, typically hydrogen and carbon[13]. Distances between two hydrogens of less than 0.5 Å can be identified by correlation with the nuclear Overhauser effect (NOE) intensities. The experimental NOE intensity and the distance between hydrogens are related by calculating both the internal motions within the molecule and its tumbling within the solvent. The slow tumbling rates in large molecules have a nominal contribution to the NOE intensity and therefore, often, only internal motions are used to approximate distances. The internal motions, or fluctuations in distances, are directly related to the intensity by $1/r^6$ where r is the distance between two protons. NOEs represent an ensemble average of all states present when collecting the NMR spectra. This makes NMR data ideal for comparison to the ensemble of structures identified by a well-sampled molecular dynamics simulation. Differences between experimental NOE distances and NOE averaged distances from MD of > 0.5 Å were considered significant.

4 Torsion Angles in Flow and Supplemental Heap Maps

In Figure 4 we present plots of torsion angles versus simulation time of $200ns$ for all glycosidic linkages in $Man_6GlcNAc_2$. All plots correspond to the flow trajectory torsion angles calculated using the following definition: for (1-x) linkages, where $x = \{1,2,3,4\}$: $\phi = H_1 - C_1 - O - C'_x$ and $\psi = C_1 - O - C'_x - H'_x$, and for the (1-6) linkages: $\phi = H_1 - C_1 - O - C'_6$, $\psi = C_1 - O - C'_6 - C'_5$ and $\omega = O - C'_6 - C'_5 - O'_5$.

In Figures 5 and 6, we present the remaining Ramachandran plots of ϕ vs ψ angles, colour-coded according to Boltzmann energy distribution for the 3-2, 4-3, 5-4 (Figure1 5) 6-5 and 7-5 (Figure1 6) linkages in diffusion and flow.

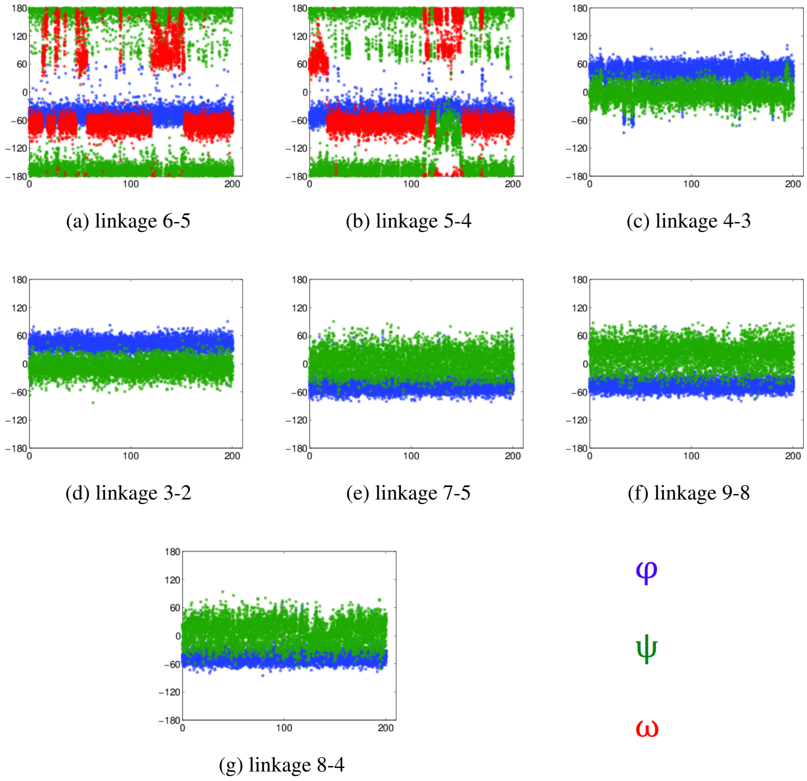

(a) linkage 6-5
(b) linkage 5-4
(c) linkage 4-3
(d) linkage 3-2
(e) linkage 7-5
(f) linkage 9-8
(g) linkage 8-4

Fig. 4: **Plots of torsion angle versus simulation time for the glycosidic linkages in flow (a) 6-5, (b) 5-4, (c) 4-3, (d) 3-2, (e) 7-5, (f) 9-8 and (g) 8-4 in $Man_6GlcNAc_2$.** Colours used in all graphs: blue for ϕ, green for ψ, and red for ω. The data were obtained from 200*ns* of unrestrained MD simulations in water.

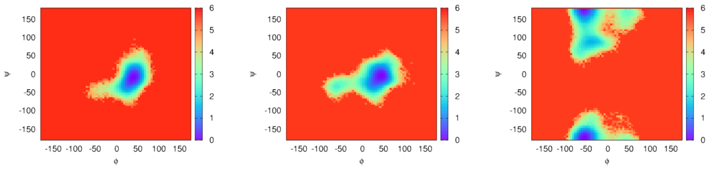

(a) 3-2 linkage - static (b) 4-3 linkage - static (c) 5-4 linkage - static

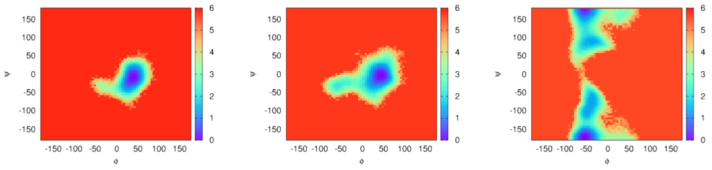

(d) 3-2 linkage - flow (e) 4-3 linkage - flow (f) 5-4 linkage - flow

Fig. 5: **Boltzmann energy heat maps for the 3-2, 4-3 and 5-4 linkages**. Ramachandran plot of ϕ vs ψ angles, colour-coded according to Boltzmann energy distribution for the 3-2 linkage in (a) static conditions and (d) flow conditions, the 4-3 linkage in (b) static conditions and (e) in flow conditions and the 5-4 linkage in (c) static conditions and (f) flow conditions. Also shown is the Ramachandran plot ψ vs ω for the 6-5 linkage. All colour bars in kcal.

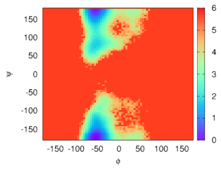

(a) 6-5 linkage - static

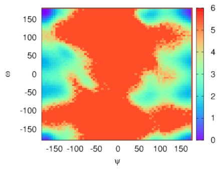

(b) 6-5 linkage - static

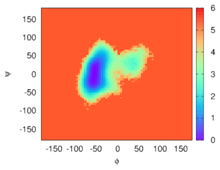

(c) 7-5 linkage - static

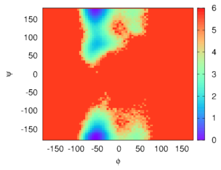

(d) 6-5 linkage - flow

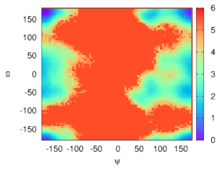

(e) 6-5 linkage - flow

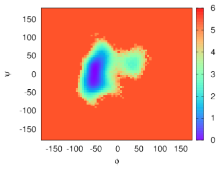

(f) 7-5 linkage - flow

Fig. 6: **Boltzmann energy heat maps for the 6-5 and 7-5 linkages**. Ramachandran plot of ϕ vs ψ angles, colour-coded according to Boltzmann energy distribution for the 6-5 linkage in (a) static conditions and (d) flow conditions. Ramachandran plot of ψ vs ω angles, colour-coded according to Boltzmann energy distribution for the 6-5 linkage in (b) static conditions and (e) flow conditions. Ramachandran plot of ϕ vs ψ angles, colour-coded according to Boltzmann energy distribution for the 7-5 linkage in (c) static conditions and (f) flow conditions. All colour bars in kcal.

References

1. Beglov, D., Roux, B.: Finite representation of an infinite bulk system: Solvent boundary potential for computer simulations. The Journal of Chemical Physics **100**(12), 9050 (1994). DOI 10.1063/1.466711. URL http://link.aip.org/link/?JCPSA6/100/9050/1
2. DeMarco, M.L., Woods, R.J.: Structural glycobiology: a game of snakes and ladders. Glycobiology **18**(6), 426–40 (2008). DOI 10.1093/glycob/cwn026. URL http://www.ncbi.nlm.nih.gov/pubmed/18390826
3. Eriksson, M., Lindhorst, T.K., Hartke, B.: Differential effects of oligosaccharides on the hydration of simple cations. The Journal of Chemical Physics **128**(10), 105,105 (2008). DOI 10.1063/1.2873147. URL http://www.ncbi.nlm.nih.gov/pubmed/18345929
4. Foley, B.L., Tessier, M.B., Woods, R.J.: Carbohydrate force fields. Wiley Interdisciplinary Reviews: Computational Molecular Science **2**(4), 652–697 (2012). DOI 10.1002/wcms.89. URL http://doi.wiley.com/10.1002/wcms.89
5. Kirschner, K.N., Yongye, A.B., Tschampel, S.M., Gonza, J., Daniels, C.R., Foley, B.L., Woods, R.J.: GLYCAM06 : A Generalizable Biomolecular Force Field. Carbohydrates. Journal of Computational Chemistry (2007). DOI 10.1002/jcc
6. Wang, J., Cieplak, P., Kollman, P.A.: How well does a restrained electrostatic potential (RESP) model perform in calculating conformational energies of organic and biological molecules? Journal of Computational Chemistry **21**(12), 1049 (2000). DOI 10.1002/1096-987X(200009)21:12¡1049::AID-JCC3¿3.3.CO;2-6. URL http://doi.wiley.com/10.1002/1096-987X(200009)21:12%3C1049::AID-JCC3%3E3.3.CO;2-6
